# Effect of continuity of care combined with family empowerment on the treatment of childhood asthma

**DOI:** 10.1097/MD.0000000000047619

**Published:** 2026-02-13

**Authors:** Mengjiao Yang, Yunsha Kong, Changna Fu, Chunling Shen, Jiahuan Wang

**Affiliations:** a Blood Collection Room, Shijiazhuang Maternal and Child Health Hospital, Shijiazhuang, Hebei Province, China; b Pediatric Surgery, Shijiazhuang Maternal and Child Health Hospital, Shijiazhuang, Hebei Province, China.

**Keywords:** adherence, asthma, children, continuity of care, family empowerment, quality of life

## Abstract

This study aims to evaluate the effectiveness of continuity of care combined with family empowerment in managing childhood asthma and provide a reference for optimizing long-term pediatric asthma management. A total of 120 children with asthma treated in our hospital from January 2022 to December 2024 were enrolled and divided into an observation group and a control group (60 cases each). The control group received routine drug therapy and basic health education. The observation group additionally received continuity of care combined with family empowerment, including establishing health records, regular telephone and home follow-ups, individualized guidance on medication and lifestyle, asthma health education, and parent empowerment training. The 2 groups were compared in terms of medication adherence, asthma control, acute attacks, emergency visits, readmission rate, quality of life (pediatric asthma quality of life questionnaire), parental empowerment (family empowerment scale), and nursing satisfaction. Baseline characteristics did not differ significantly between groups (*P* > .05). After intervention, the observation group showed significantly better medication adherence than the control group (regular medication 88.3% vs 68.3%, correct inhaler use 83.3% vs 63.3%, no missed or reduced doses 86.7% vs 65.0%; all *P* < .05). The complete asthma control rate was higher (56.7% vs 33.3%; *P* = .015), while the 6-month frequency of acute attacks (1.2 ± 0.9 vs 2.0 ± 1.1), emergency visits (13.3% vs 30.0%), and readmissions (8.3% vs 25.0%) were lower (all *P* < .05). The pediatric asthma quality of life questionnaire scores improved more in the observation group across all dimensions (*t* = 6.32–7.44; *P* < .001), and family empowerment scale scores for family management, communication, and self-efficacy were also significantly higher (*t* = 6.02–9.42; *P* < .001). Nursing satisfaction was greater in the observation group (93.3% vs 75.0%; *P* = .015). Continuity of care combined with family empowerment significantly enhances medication adherence and asthma control, reduces exacerbations and readmissions, improves children’s quality of life, and strengthens parents’ management ability and self-efficacy. This integrated model is valuable for the long-term management of childhood asthma.

## 1. Introduction

Bronchial asthma is one of the most common chronic respiratory diseases among children worldwide. Its main pathological features are chronic airway inflammation and hyperresponsiveness, with clinical manifestations including recurrent wheezing, cough, chest tightness, and dyspnea. According to the latest report of the Global Initiative for Asthma, the prevalence of childhood asthma continues to rise, making it an important public health issue that affects children’s health, quality of life, and social functioning.^[1,2]^ In China, with the acceleration of industrialization and urbanization, environmental pollution, lifestyle changes, and increased allergen exposure have contributed to the yearly rise in asthma incidence among children. Epidemiological data indicate that the prevalence of childhood asthma in China has reached 3% to 7%, with a trend toward younger onset.^[3,4]^ Asthma not only compromises children’s physical health but also seriously impacts their psychological development and social adaptability, increases family financial burden, and has become a major health challenge in pediatrics requiring urgent attention.

Asthma is a typical chronic disease, and the key goal of treatment lies in long-term effective control rather than short-term relief. Standardized pharmacological treatments (such as inhaled corticosteroids) can significantly improve symptoms and lung function. However, in the absence of long-term standardized management, children are prone to poor disease control because of inadequate adherence, insufficient family care, and ineffective communication between physicians and patients.^[5]^ Previous studies have shown that about 30% to 50% of children with asthma have poor medication adherence. Some parents lack a comprehensive understanding of the disease and fail to recognize triggers and early warning signs of exacerbations in a timely manner, thereby increasing the risk of acute attacks and repeated hospitalizations.^[6]^ Traditional discharge health education mostly remains at the level of “1-time guidance,” lacking continuity and individualization, which makes it difficult to meet the long-term needs of chronic disease management.

Continuity of care is a new model that has been promoted in chronic disease management nursing in recent years, emphasizing the linkage and extension of inhospital and out-of-hospital care.^[7]^ Through telephone follow-up, online platform management, and home visits, nurses can continuously monitor changes in the child’s condition, provide individualized health education, and dynamically adjust care plans based on feedback, thereby improving medication adherence and disease control levels.^[8,9]^ Studies at home and abroad have confirmed that continuity of care not only reduces the readmission rate of patients with chronic diseases but also significantly improves the quality of life of patients and their families.

At the same time, the theory of family empowerment has gradually been applied to the management of pediatric chronic diseases. This theory emphasizes enhancing parents’ confidence and ability in disease management through knowledge transfer, skills training, and psychological support, transforming them from “passive recipients” to “active participants.”^[10]^ Research has shown that family empowerment interventions can significantly improve parents’ disease-related knowledge and self-efficacy, promote effective communication between physicians and families, and thereby improve the overall quality of asthma management in children.^[11]^ For children with asthma, who are highly dependent on parental care, the value of family empowerment is particularly important.

Therefore, integrating continuity of care with family empowerment can not only improve adherence through continuous follow-up and dynamic management but also enhance parents’ management ability and initiative through empowerment education. This approach is expected to achieve more significant effects in asthma control, prevention of acute attacks, improvement of quality of life, and enhancement of nursing satisfaction. Based on this, the present study aimed to explore the application effect of continuity of care combined with family empowerment in the treatment of childhood asthma, providing evidence for optimizing pediatric asthma nursing models and promoting the development of chronic disease management systems.

## 2. Data and methods

### 2.1. Research design

This retrospective study was approved by the Ethics Committee of Shijiazhuang Maternal and Child Health Hospital. Pediatric patients with bronchial asthma who attended the outpatient department of our hospital between January 2022 and December 2024 were included, with a total of 120 cases enrolled. According to the nursing models previously implemented in clinical practice, the patients were nonrandomly assigned to an observation group (n = 60) and a control group (n = 60). The control group received routine asthma medication and basic health education, while the observation group received continuity of care combined with family empowerment interventions on this basis, including postdischarge follow-up management, individualized health education, family participation in asthma management, and joint care planning by nurses and parents.

### 2.2. Inclusion and exclusion criteria

Inclusion criteria: meeting the diagnostic criteria for childhood asthma in the *Guidelines for the Diagnosis and Treatment of Childhood Bronchial Asthma*; aged 3 to14 years, regardless of sex; receiving systematic treatment in the pediatric outpatient or inpatient department of our hospital, with complete medical records and accessible follow-up information; previously receiving routine asthma medication treatment, with some patients having undergone continuity of care and family empowerment interventions during treatment; parents or primary caregivers with basic communication skills who were willing to actively cooperate with relevant nursing and health education.

Exclusion criteria: combined with other severe respiratory diseases (such as congenital pulmonary malformations, bronchiectasis, chronic obstructive pulmonary disease, etc); severe dysfunction of vital organs such as the heart, liver, or kidneys, or immune system diseases; history of severe psychiatric or neurological disorders affecting adherence and communication; incomplete medical records, or missing/lost follow-up data; parents refusing or unable to cooperate with continuity of care and family empowerment interventions.

## 3. Nursing methods

All enrolled children received a standardized medication regimen during hospitalization, and all met the basic disease control criteria at discharge.

### 3.1. Control group

At discharge, routine health guidance was provided by the responsible nurse, including daily medication use for asthma, coping measures during attacks, and basic precautions. No further systematic nursing follow-up intervention was performed thereafter.

### 3.2. Observation group

On the basis of the routine discharge guidance given to the control group, continuity of care combined with family empowerment interventions was implemented. The specific contents were as follows:

#### 3.2.1. Intervention methods

After discharge, the responsible nurse established a health record for each child and maintained communication with parents through telephone follow-ups, WeChat/QQ group exchanges, and home visits. At least 1 telephone follow-up was conducted per month, and 1 home visit every 2 months, with continuous intervention for 6 months. Nursing priorities were dynamically adjusted based on parental feedback during follow-ups.

#### 3.2.2. Family continuity of care contents

*Medication management:* Individualized medication guidance was provided according to the educational level, economic status, and adherence characteristics of different families. Parents were given detailed explanations of the mechanisms of action, administration methods (especially correct use of inhalation devices), dosages, and precautions of commonly used drugs, emphasizing that doses should not be altered arbitrarily. During follow-ups, nurses checked and supervised the children’s medication use to improve adherence.

*Lifestyle management:* Parents were educated on asthma-related medical knowledge, assisted in identifying common triggers (such as allergens, climate changes, strenuous exercise, etc) and early warning signs of attacks, and guided to reasonably avoid risks. Children were encouraged to maintain good daily routines, participate appropriately in moderate activities such as swimming, jogging, and gymnastics, while avoiding overly strenuous exercise. Dietary guidance emphasized balanced nutrition, increased intake of fruits and vegetables, and avoidance of cold drinks and spicy or irritating foods. In daily life, attention was paid to keeping warm and reducing exposure to environmental allergens.

*Disease education and empowerment:* Through health manuals and educational materials, the concept of chronic disease management of asthma was systematically explained, stressing the importance of standardized long-term treatment. Parents were trained to master family self-monitoring skills, such as peak flow measurement and symptom diary recording, to enhance their ability for early recognition and management. During each follow-up, nurses asked questions and collected feedback based on the previous education focus, identifying knowledge gaps and providing targeted reeducation, thereby gradually strengthening the self-management ability of both parents and children.

### 3.3. Data collection

A total of 120 children with asthma who received systematic treatment in the pediatric outpatient and inpatient departments of our hospital from January 2022 to December 2024 were included in this study. The data collected were as follows:

1. General information: basic data such as age, sex, disease duration, family history, and allergy history were collected through the medical record system and admission records.
2. Medication adherence: during follow-up, the responsible nurse collected information on regular medication use, correct use of inhalation devices, and whether there were missed doses or arbitrary dose reductions, based on parental interviews, children’s medication diaries, and checking remaining medications. The results were calculated as percentages.
3. Asthma control level: according to the Global Initiative for Asthma criteria, asthma control levels were classified as complete control, partial control, and uncontrolled. Data were determined comprehensively through outpatient follow-up, parental feedback, and medical records.
4. Number of attacks and emergency/readmission status: the number of acute attacks within 6 months of intervention was recorded by the follow-up nurse, while information on emergency visits and readmissions was collected through the hospital information system and parental interviews.
5. Quality of life (pediatric asthma quality of life questionnaire [PAQLQ]): The PAQLQ, which includes 3 dimensions (activity limitation, symptoms, and emotional function), was used. The children completed the questionnaire under the guidance of nurses, and assessments were conducted before intervention and 6 months after intervention.
6. Parental empowerment level (family empowerment scale [FES]): The FES, which includes family management ability, communication with healthcare providers, and self-efficacy, was used. The child’s primary caregiver completed the questionnaire before intervention and 6 months after intervention, and all questionnaires were collected uniformly by nurses.
7. Nursing satisfaction: a nursing service satisfaction questionnaire designed by our hospital was used, covering service attitude, professional competence, and communication skills. It was divided into 4 levels: very satisfied, satisfied, average, and dissatisfied. Parents filled in the questionnaire anonymously at the end of the intervention.

### 3.4. Statistical analysis

All data were statistically analyzed using SPSS 26.0 software (Armonk). Measurement data (such as age, disease duration, number of attacks, PAQLQ scores, and FES scores) were tested for normality and expressed as mean ± standard deviation ($\bar{x}\pm s$). Independent sample *t*-tests were used for between-group comparisons, and paired *t*-tests for within-group pre- and postintervention comparisons; if normal distribution was not met, nonparametric rank-sum tests were used. Categorical data (such as sex distribution, family history, allergy history, medication adherence, asthma control level, emergency visit and readmission rates, and nursing satisfaction) were expressed as frequency and percentage (n/%), and between-group comparisons were performed using the *χ*^2^ test or Fisher’s exact probability method. All tests were 2-tailed, and *P* < .05 was considered statistically significant.

## 4. Results

### 4.1. Comparison of general data

A total of 120 children with asthma were included, with 60 cases each in the observation and control groups. The mean ages were 7.8 ± 2.6 and 7.3 ± 2.9 years, respectively; the male-to-female ratios were 37:23 and 33:27. The distributions of disease duration, family history, allergy history, and asthma severity (mild/moderate/severe: 28/25/7 vs 30/23/7) were comparable between groups, with no statistically significant differences (all *P* > .05). These results indicate good baseline comparability for subsequent analysis Table 1.

**Table 1:** Comparison of baseline characteristics between the 2 groups ($\bar{x}\pm \;s$, n/%).

Variable	Observation group (n = 60)	Control group (n = 60)	Statistic	*P* value
Age (yr)	7.8 ± 2.6	7.3 ± 2.9	*t* = 1.02	.31
Gender (male/female)	37 (61.7%)/23 (38.3%)	33 (55.0%)/27 (45.0%)	*χ*^2^ = 0.56	.453
Disease course (yr)	3.1 ± 1.5	2.8 ± 1.6	*t* = 1.01	.315
Family history (yes/no)	21 (35.0%)/39 (65.0%)	17 (28.3%)/43 (71.7%)	*χ*^2^ = 0.59	.442
Allergy history (yes/no)	28 (46.7%)/32 (53.3%)	24 (40.0%)/36 (60.0%)	*χ*^2^ = 0.57	.449
Asthma severity (mild/moderate/severe)	28 (46.7%)/25 (41.7%)/7 (11.6%)	30 (50.0%)/23 (38.3%)/7 (11.7%)	*χ*^2^ = 0.18	.913

### 4.2. Comparison of medication adherence

In the observation group, 53 (88.3%) cases demonstrated regular medication use, which was significantly higher than 41 (68.3%) cases in the control group, with a statistically significant difference (*χ*^2^ = 6.77, *P* = .009). Regarding the correct use of inhalation devices, 50 (83.3%) cases in the observation group were correct compared with 38 (63.3%) cases in the control group, and the difference was statistically significant (*χ*^2^ = 6.12, *P* = .013) (Table 2). In addition, 52 (86.7%) cases in the observation group reported no missed doses or arbitrary dose reductions, which was also significantly higher than 39 (65.0%) cases in the control group (*χ*^2^ = 7.12, *P* = .008).

**Table 2:** Comparison of medication adherence (n/%).

Indicator	Observation group (n = 60)	Control group (n = 60)	*χ*^2^ value	*P* value
Regular medication use	53 (88.3%)	41 (68.3%)	6.77	.009
Correct inhaler technique	50 (83.3%)	38 (63.3%)	6.12	.013
No missed/reduced doses	52 (86.7%)	39 (65.0%)	7.12	.008

### 4.3. Comparison of asthma control levels

After 6 months of intervention, in the observation group, 34 (56.7%) cases achieved complete control, 20 (33.3%) cases partial control, and 6 cases (10.0%) were uncontrolled; in the control group, the corresponding numbers were 20 (33.3%) cases, 23 (38.3%) cases, and 17 (28.3%) cases, respectively. The distribution of asthma control levels between the 2 groups showed a statistically significant difference (*χ*^2^ = 8.41, *P* = .015) (Table 3).

**Table 3:** Asthma control level based on GINA (n/%).

Control level	Observation group (n = 60)	Control group (n = 60)	*χ*^2^ value	*P* value
Well controlled	34 (56.7%)	20 (33.3%)		
Partly controlled	20 (33.3%)	23 (38.3%)		
Uncontrolled	6 (10.0%)	17 (28.3%)	8.41	.015

GINA = global initiative for asthma.

### 4.4. Comparison of the number of attacks and emergency/readmission rates

After 6 months of intervention, the average number of acute attacks in the observation group was 1.2 ± 0.9, which was significantly lower than 2.0 ± 1.1 in the control group (*t* = 4.09, *P* < .001). There were 8 cases of emergency visits (13.3%) in the observation group, lower than 18 (30.0%) cases in the control group (*χ*^2^ = 4.63, *P* = .031); there were 5 cases of readmission (8.3%) in the observation group, also significantly fewer than 15 (25.0%) cases in the control group (*χ*^2^ = 6.07, *P* = .014) (Table 4).

**Table 4:** Frequency of exacerbations, emergency visits, and readmission ($\bar{x}\pm \;s$, n/%).

Indicator	Observation group (n = 60)	Control group (n = 60)	Statistic	*P* value
Acute exacerbations (times/6 mo)	1.2 ± 0.9	2.0 ± 1.1	*t* = 4.09	<.001
Emergency visit rate	8 (13.3%)	18 (30.0%)	*χ*^2^ = 4.63	.031
Readmission rate	5 (8.3%)	15 (25.0%)	*χ*^2^ = 6.07	.014

### 4.5. Comparison of quality of life (PAQLQ)

After the intervention, the PAQLQ scores of both groups improved significantly compared with those before the intervention (*P* < .001) (Table 5). Among them, the observation group showed significantly greater improvements than the control group in activity limitation, symptoms, emotional function, and total score (*t* = 6.32–7.44, all *P* < .001), indicating that continuity of care combined with family empowerment intervention can effectively improve the quality of life of children with asthma.

**Table 5:** Comparison of quality of life scores (PAQLQ, $\bar{x}\pm \;s$).

Dimension	Observation pre	Observation post	*t* (Within)	*P* (within)	Control pre	Control post	*t* (Within)	*P* (within)	*t* (between, post)	*P* (between, post)
Activity	4.1 ± 0.8	5.8 ± 0.7	12.3	<.001	4.0 ± 0.9	4.9 ± 0.8	6.8	<.001	6.32	<.001
Symptoms	3.9 ± 0.7	5.7 ± 0.6	13.1	<.001	3.8 ± 0.8	4.8 ± 0.7	7.9	<.001	7.01	<.001
Emotional function	4.2 ± 0.9	6.0 ± 0.6	12.8	<.001	4.1 ± 0.8	5.0 ± 0.7	7.1	<.001	7.44	<.001
Total score	4.1 ± 0.7	5.8 ± 0.6	14.2	<.001	4.0 ± 0.8	4.9 ± 0.7	7.6	<.001	7.12	<.001

PAQLQ = pediatric asthma quality of life questionnaire.

### 4.6. Comparison of parental empowerment levels

After the intervention, the FES scores of parents in both groups, including each dimension and the total score, were significantly improved compared with those before the intervention (*P* < .001) (Table 6). Among them, the observation group showed significantly greater improvements than the control group in family management, communication with healthcare providers, self-efficacy, and total score (*t* = 6.02–9.42, all *P* < .001), indicating that continuity of care combined with family empowerment intervention can effectively enhance parents’ management ability and self-efficacy.

**Table 6:** Comparison of parental empowerment scores (FES, $\bar{x}\pm \;s$).

Dimension	Obs. pre	Obs. post	*t* (Within)	*P* (within)	Ctrl. pre	Ctrl. post	*t* (Within)	*P* (within)	*t* (Between, post)	*P* (between, post)
Family management	55.2 ± 6.8	72.5 ± 5.9	12.8	<.001	54.7 ± 7.1	64.3 ± 6.4	7.3	<.001	7.18	<.001
Communication with staff	28.4 ± 4.2	38.9 ± 3.7	12.2	<.001	28.1 ± 4.5	33.5 ± 3.9	6.5	<.001	6.02	<.001
Self-efficacy	21.7 ± 3.8	30.6 ± 3.2	11.6	<.001	21.4 ± 3.9	25.8 ± 3.5	6.9	<.001	7.01	<.001
Total score	105.3 ± 9.5	142.0 ± 8.2	14.9	<.001	104.2 ± 9.8	123.6 ± 8.7	8.2	<.001	9.42	<.001

FES = family empowerment scale.

### 4.7. Comparison of nursing satisfaction

In the observation group, the proportion of parents who were “very satisfied” or “satisfied” with nursing care was 93.3% (56/60), which was significantly higher than 75.0% (45/60) in the control group. In the control group, the proportion of “dissatisfied” cases was 8.3% (5/60), while no cases of dissatisfaction were reported in the observation group (Table 7). The difference in nursing satisfaction between the 2 groups was statistically significant (*χ*^2^ = 10.42, *P* = .015).

**Table 7:** Comparison of nursing satisfaction (n/%).

Satisfaction level	Observation group (n = 60)	Control group (n = 60)	*χ*^2^ value	*P* value
Very satisfied	39 (65.0%)	25 (41.7%)		
Satisfied	17 (28.3%)	20 (33.3%)		
Fair	4 (6.7%)	10 (16.7%)		
Dissatisfied	0 (0.0%)	5 (8.3%)	10.42	.015

## 5. Discussion

The results of this study showed that continuity of care combined with family empowerment intervention has significant advantages in the management of childhood asthma. Compared with the control group, the observation group demonstrated notable improvements in medication adherence, asthma control level, and frequency of acute attacks, emergency visit and readmission rates, quality of life, parental empowerment levels, and nursing satisfaction. These findings suggest that integrating continuity of care with family empowerment is an effective approach to optimizing long-term management of childhood asthma.

First, in terms of medication adherence, the observation group achieved significantly higher rates of regular medication use, correct inhalation device operation, and absence of missed or arbitrarily reduced doses compared with the control group. This result is consistent with previous studies, as continuity of care, through telephone calls, WeChat communication, and home visits, strengthens continuous monitoring and feedback by healthcare providers for families, enabling timely identification and correction of poor adherence.^[12]^ At the same time, family empowerment allows parents to fully understand the mechanisms of action and importance of standardized medication use, enhancing their sense of responsibility and initiative in medication management, thereby reducing adverse behaviors caused by misunderstanding or negligence. This sustained 2-way communication and educational mechanism provides a safeguard for improving medication adherence.^[13]^

Regarding asthma control, the proportion of children with complete control was significantly higher, and the proportion with uncontrolled asthma was markedly lower in the observation group compared with the control group. This indicates that the intervention effectively improves long-term asthma management. The underlying mechanisms may include 2 aspects: on the 1 hand, continuity of care, through individualized health education and dynamic follow-up, helps families promptly recognize asthma triggers and early symptoms, thereby reducing acute attacks^[14]^; on the other hand, family empowerment enhances parents’ self-monitoring and coping skills, enabling them to take earlier intervention measures, such as appropriate use of reliever medications or environmental adjustments, thereby maintaining a higher level of disease control.^[15]^

In terms of the number of acute attacks and readmissions, this study found that the observation group had significantly fewer attacks, emergency visits, and readmissions than the control group. The clinical significance of this result is particularly noteworthy, as reducing acute attacks and readmissions not only alleviates the suffering of children but also substantially lowers the medical burden on families and society. Continuity of care improves treatment continuity through follow-up and supervision, preventing disease deterioration caused by treatment interruptions^[16]^; meanwhile, family empowerment enables parents to take action quickly in the early stages of an attack, effectively preventing exacerbation. This collaborative model between healthcare providers and families establishes a forward-shifted defense mechanism, fundamentally reducing the risk of recurrent acute asthma attacks.^[17]^

Regarding quality of life, PAQLQ assessments showed that both groups improved after the intervention, but the observation group made more significant progress, particularly in activity limitation, symptom control, and emotional function. The impact of asthma on children extends beyond physical symptoms, also involving activity restrictions, barriers to learning participation, and emotional distress. Continuity of care, through individualized exercise and lifestyle guidance, helps children gradually restore and maintain appropriate activity levels^[18]^; at the same time, family empowerment enhances parents’ understanding of the disease and confidence in management, allowing children to gain more psychological support and encouragement. These combined factors improve both the daily life experience and psychological well-being of children.^[19]^

These findings are consistent with the principles of the family-centered care model widely adopted in the United States, which emphasizes partnership, respect, and shared decision-making between healthcare professionals and families.^[20]^ Similar to family-centered care, our combined approach of continuity of care and family empowerment promotes parents’ participation in care planning and strengthens their confidence in disease management. Furthermore, the intervention aligns with the self-management education programs implemented in the United Kingdom, which aim to enhance patients’ and caregivers’ self-efficacy through structured education and follow-up.^[19]^ However, unlike these well-established models, our program integrates hospital–community continuity with family empowerment tailored to local healthcare resources, offering a more practical framework for pediatric asthma management in China.

In addition, results from the FES indicated that the observation group showed significant improvements in family management ability, communication with healthcare providers, and self-efficacy. Parents play a central role in the management of childhood asthma, and their knowledge, communication skills, and sense of self-efficacy directly affect disease control outcomes. Continuity of care provides parents with a continuous platform for learning and feedback, while empowerment education helps parents shift from passive implementers to active decision-makers.^[20]^ This transformation not only strengthens parents’ ability to manage their children within the home environment but also fosters a cooperative relationship between families and healthcare providers, forming positive interactions.

The nursing satisfaction results further confirmed the feasibility and acceptability of this intervention model. Parents in the observation group reported significantly higher satisfaction with nursing care than those in the control group, reflecting the positive role of continuity of care and family empowerment in improving service quality, promoting communication, and enhancing parental participation.^[21]^ Increased satisfaction contributes to stronger family recognition and cooperation with the nursing model, thereby further consolidating intervention effects.

This study confirmed the clinical value of continuity of care combined with family empowerment from multiple perspectives. The underlying mechanism lies in establishing a continuous chain of in-hospital and out-of-hospital management through “continuity of care,” ensuring that treatment and nursing do not end with discharge, and enhancing parental initiative and self-efficacy in the management process through “family empowerment,” thereby integrating nursing interventions into the family and daily life. This model of “professional guidance + family participation” compensates for the shortcomings of traditional nursing and embodies the dual responsibility of healthcare providers and families in chronic disease management.^[19–21]^

Of course, this study has certain limitations. The sample size was relatively small, and the intervention period was only 6 months; thus, the long-term effects require further follow-up. In addition, as this was a retrospective study with nonrandom grouping based on historical nursing models, potential selection bias cannot be completely ruled out, which may affect the comparability of the groups. Although asthma severity has been included as a baseline variable to reduce bias, information on other potential confounding factors, such as parents’ education level and family economic status, was not consistently available in the medical records and therefore could not be analyzed, which may have introduced residual confounding. Moreover, the generalizability of this intervention needs to be further verified across different regions and healthcare settings. Future studies should adopt prospective randomized controlled designs and incorporate digital follow-up systems or telemedicine platforms to enhance the continuity, accessibility, and effectiveness of care.

In conclusion, continuity of care combined with family empowerment can significantly improve medication adherence and disease control in children with asthma, reduce acute attacks and readmissions, enhance quality of life and parental management ability, and achieve higher nursing satisfaction, demonstrating strong clinical value for promotion.

## Author contributions

**Conceptualization:** Mengjiao Yang, Yunsha Kong, Jiahuan Wang.

**Data curation:** Mengjiao Yang, Yunsha Kong, Changna Fu, Jiahuan Wang.

**Formal analysis:** Mengjiao Yang, Yunsha Kong, Jiahuan Wang.

**Funding acquisition:** Mengjiao Yang, Changna Fu, Jiahuan Wang.

**Investigation:** Jiahuan Wang.

**Writing – original draft:** Mengjiao Yang, Yunsha Kong, Chunling Shen, Jiahuan Wang.

**Writing – review & editing:** Mengjiao Yang, Yunsha Kong, Chunling Shen, Jiahuan Wang.
